# User Behavior Shift Detection in Ambient Assisted Living Environments

**DOI:** 10.2196/mhealth.2536

**Published:** 2013-06-18

**Authors:** Asier Aztiria, Golnaz Farhadi, Hamid Aghajan

**Affiliations:** ^1^University of MondragonMondragonSpain; ^2^Stanford UniversityStanford, CAUnited States

**Keywords:** shift detection, intelligent environments, disease detection

## Abstract

Identifying users’ frequent behaviors is considered a key step to achieving real, intelligent environments that support people in their daily lives. These patterns can be used in many different applications. An algorithm that compares current behaviors of users with previously discovered frequent behaviors has been developed. In addition, it identifies the differences between both behaviors. Identified shifts can be used not only to adapt frequent behaviors, but also shifts may indicate initial signs of some diseases linked to behavioral modifications, such as depression or Alzheimer’s. The algorithm was validated using datasets collected from smart apartments where five different ADLs (Activities of Daily Living) were recognized. It was able to identify all shifts from frequent behaviors, as well as identifying necessary modifications in all cases.

## Introduction

Ubiquitous computing [1] refers to a paradigm in which a new type of relation between users and technology is established. It provides a widespread and transparent technology to the user. Modern computing devices of various types are ubiquitous, embedded in different objects that users can interact with and that consequently influence users’ lifestyles. An important further development of this concept has resulted in concepts including intelligent environments [2], ambient intelligence (AmI) [3], smart environments [4], pervasive computing [5], and Ambient Assisted Living (AAL) [6]. These refer to digital environments that proactively, but sensibly, support people in their daily lives [7]. Being sensible demands recognizing the user, learning or knowing her/his preferences and, given the current situation, acting in accordance with it. Several systems have been developed for learning users’ frequent behaviors without disturbing them, ie, in a transparent way. The Learning Frequent Patterns of User Behavior System (LFPUBS) [8] takes as a starting point the data collected from sensors, discovers users’ frequent behaviors, and represents such patterns in a comprehensible way.

Once frequent behaviors of a user have been discovered, depending on the needs and situation of that specific user, such patterns can be used for many different purposes. An important application is behavior shift detection by comparing the current user’s behavior with the previously discovered frequent behaviors. A shift from the frequent behaviors is not necessarily abnormal. This is because users can change their behaviors over time. Nevertheless, these shifts provide valuable information to the environment because they:

- can show initial signs of some diseases (eg, Alzheimer’s disease, depression) or the beginning of unhealthy habits
- can be very helpful to confirm disease diagnoses, eg, the environment can record historical data about shifts so that experts in the domain can use them as additional information
- can show change of preferences, ie, users can change their frequent behaviors for several reasons and therefore, the environment should adapt the patterns based on the user behavior shifts

This paper develops an algorithm that compares the current behavior of a user with previously discovered frequent behaviors and identifies shifts. In addition, the proposed algorithm determines the criticality of different shifts for certain users and specific applications.

### Related Work

Understanding human behavior has attracted a significant number of researchers, and much work has been devoted to modeling human behavior in order to act accordingly. Mozer et al [9] and Chang et al [10] were among the first to report on applications for ambient intelligence environments where user patterns were considered. Based on residents’ lifestyles, models to predict occupancy were created. The models then were used for lighting control. Several other methods for identifying users’ patterns have been proposed [11,12]. Holistic approaches considering the special features of intelligent environments have been also investigated [8]. A survey of these methods is given in Aztiria et al [13].

Due to the novelty and characteristics of intelligent environments, complex model-based applications have not been developed. Artificial Intelligence techniques in relation to Alzheimer’s disease have been used. Zhou et al [14] used multitask regression models in order to track markers that allow identification of the progression of the disease. Bouchard et al [15] developed a hybrid plan recognition model, based on probabilistic description logic, which addressed the issue of recognizing the activities and errors of Alzheimer’s patients.

Shifts in human behaviors have been analyzed in many domains such as Web navigation and activity workflow [16,17]. However, it is necessary to obtain a specific solution taking into account the special features of intelligent environments, such as importance of the user, transparency, nature of collected data, etc.

## Methods

### General Architecture

**Figure 1:**
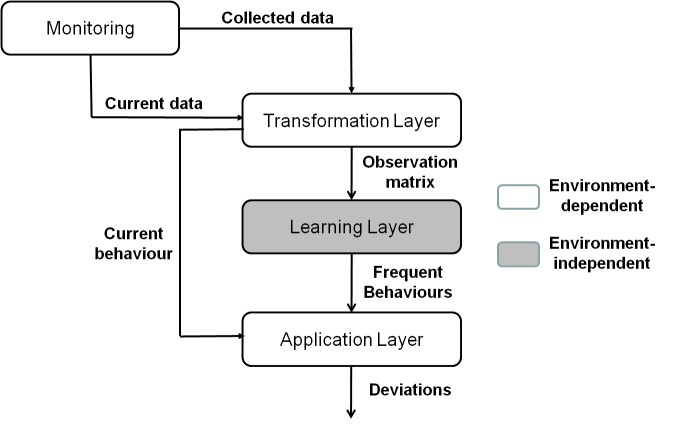
General architecture for identifying shifts.

Identifying shifts involves several steps as shown in Figure 1. The first step is environment monitoring for collecting data. This monitoring task should be carried out as unobtrusively as possible. The next step is to infer meaningful information from the collected data. The objective of the transformation layer is to identify actions defined as interesting. The set of actions to be identified is denoted by A={ai}. The output of this layer is stored in the observation matrix, X. The observation matrix represents the occurrences of different actions, ai ∈ A, in different timestamps.

Given the observation matrix, the learning layer discovers the set of frequent behaviors. Frequent behaviors are obtained using the LFPUBS method given by Aztiria [8]. LFPUBS first discovers the set of actions that frequently occur together and then it identifies the order of such actions, defining each frequent behavior as a Markov chain.

Let F denotes the set of frequent behaviors of a user. Then fi ∈ F is a set of actions, {fi_k_ : fi_k_ ∈ A}, that forms a Markov chain with initial probability, P_0_=Pr(fi_0_ ) and the transition matrix P=[Pk,j] where Pk,j=Pr(fi_j_ |fi_k_ ). Apart from this definition, LFPUBS can discover time relationship between different actions of the model (eg, Michael needs approximately 10 minutes to go to the bathroom) and identify under what conditions this pattern occurs (eg, under what conditions Michael has a shower or not).

The following scenario exemplifies the common behavior of a user. On weekdays, Michael’s alarm clock goes off (‘Alarm, on’) a few minutes after 08:00AM. Approximately 10 minutes after getting up, he usually steps into the bathroom (‘Bathroom, on’), and sometimes he takes a shower (‘Shower, on’) and some other times he does not. Then, he goes to the kitchen (‘Kitchen, on’), and after having breakfast (‘Breakfast, on’), he takes his daily pill (‘Pill, on’).

**Figure 2:**
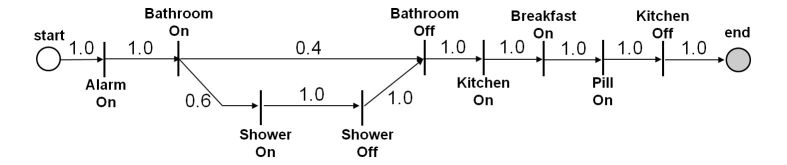
Michael's morning ritual represented in a Markov Chain.

Michael’s morning behavior is discovered by the learning algorithm and represented as a Markov chain as shown in Figure 2. The transition probabilities shown in the chain indicate the frequency of that relationship.

Finally, the application layer allows the development of applications that can benefit from the discovered frequent behaviors. This paper proposes an algorithm for shift detection in user behavior, which compares the current set of observed actions from the user, C={ci : ci ∈ A}, with all frequent behaviors, fi ∈ F, obtained in the learning layer. If the current behavior matches any of the frequent behaviors, the algorithm returns a likelihood value. Otherwise, the algorithm calculates the shift of the current behavior, C, from all frequent behaviors and determines the criticality of these differences (see Textbox 1.

**Identifying Shifts From Frequent Behaviors.**

**Algorithm**: calculate Shifts (F, C)

**Input**: Set of frequent behaviors F and current behavior C.

**Output**: Likelihood (LL), number of modifications, and criticality.

for each fi ∈ F compute LL(C|fi)

   if LL(C|fi) != 0 then return LL(C|fi)

   else

      calculateAllPossiblePaths(fi)

      for each path ρij ∈ fi

         calculateNecessaryModifications (ρij ,C)

         return numberModification, modificationsCriticality

#### Calculating Likelihoods

Given the current behavior, C, the first step is to determine if that behavior is part of user frequent behaviors, F. This requires comparing the current behavior with all previously discovered frequent behaviors fi ∈ F ∀i. Recall that each frequent behavior fi is represented as a Markov chain. Then, the likelihood is obtained as in Equation 1 in Multimedia Appendix 1, where |·| denotes the cardinality of the set and Pr (ck → ck+1) is given by the transition probability matrix for the frequent behavior fi. In general, the likelihood value implies the frequency of that behavior. On the other hand, having LL(C|fi)=0 ∀fi ∈ F indicates the current behavior is not frequent.

For example, consider the following three current behaviors and the transition matrix defined by the likelihoods shown by Figure 2:

C1 = ‘Alarm, on’, ‘Bathroom, on’, ‘Shower, on’, ‘Shower off’, ‘Bathroom off’ ‘Kitchen, on’, ‘Breakfast, on’, ‘Pill, on’, ‘Kitchen off’

C2 = ‘Alarm, on’, ‘Bathroom, on’, ‘Shower, on’, ‘Shower off’, ‘Bathroom off’ ‘Kitchen, on’, ‘Breakfast, on’, ‘Kitchen off’

C3 = ‘Alarm, on’, ‘Bathroom, on’, ‘Shower, on’, ‘Shower off’, ‘Bathroom off’ ‘Kitchen, on’, ‘Pill, on’, ‘Breakfast, on’, ‘Kitchen off’

Then, LL(C1|f1)=0.6, LL(C2|f1)= LL(C3|f1) = 0.

However, note that having a behavior that differs from frequent behaviors (ie, zero likelihood) is going to be common. For example, in Michael’s case, although the likelihoods of current behaviors C2 and C3 are zero, it is clear that they slightly differ from the frequent behaviors. Therefore, it is necessary to determine how different a behavior is from frequent behaviors.

#### Calculating Paths

In order to be able to identify how different a current behavior is from a frequent behavior, it is necessary to calculate all the possible behaviors that can be represented by the frequent behavior. Thus, all the possible behaviors that can be represented by the frequent behavior should be obtained. Possible paths included in a frequent behavior fi ∈ F are obtained using the depth-first search algorithm [18]. In addition, the likelihood of each path serves as a criterion to discard all the behaviors whose likelihoods are below a certain threshold. This condition is necessary because loops or self loops in the Markov chain can lead to infinite numbers of paths. Let ρij = {ρij_k_ : ρij_k_ ∈ *A*} denote the set of actions over the j^th^ path for the i^th^ frequent behavior, fi ∈ F. Then, the likelihood of the path is given by Equation 2 in Multimedia Appendix 1, where Pr(ρij_k_ → ρij_k+1_ )is given by the transition probability matrix for the frequent behavior fi. Recall Michael’s morning frequent behavior, f1. It consists of two sequences of actions:

ρ11: ‘Alarm, on’, ‘Bathroom, on’, ‘Bathroom, off’, ‘Kitchen, on’, ‘Breakfast, on’,‘Pill, on’, ‘Kitchen, off’ where LL(ρ11)=0.4

ρ12: ‘Alarm, on’, ‘Bathroom, on’, ‘Shower, on’, ‘Shower, off’, ‘Bathroom, off’, ‘Kitchen, on’, ‘Breakfast, on’,‘Pill, on’,‘Kitchen, off’ where LL(ρ12)=0.6

#### Calculating Modifications and Criticality

Once all possible paths have been calculated, the next step is to compare C with all them. It is worth remembering that in order to reach this point, the likelihood (C|fi) must be 0.0, so that it needs some modifications to match any of the paths. For that, C is compared to all the paths of fi and needed modifications are calculated, as well as their criticality.

Recall that for LL (C|fi) = 0, ∀fi ∈ F, the algorithm should obtain the minimum number of modifications that matches the current behavior with any fi ∈ F. For each fi ∈ F, the algorithm compares the current behavior, C, with all the paths, ρij, and obtains the set of modifications as well as the corresponding criticality values.

##### Identifying Modifications

The process to identify modifications is an adaptation of the Levenshtein distance [19]. Given two sequences of actions, C and ρij, it calculates the set of modifications in C to get ρij. In intelligent environments, the set of all possible modifications denoted by H is the union of:

- H1= {*insert* (ai): ∀ai ∈ A}: Insertion of an action if the user forgets to do an action.
- H2= {*delete* (ai): ∀ai ∈ A}: Deletion of an action if the user does an extra action.
- H3= {*subs* (ai,aj): ∀ai,aj ∈ A}: Substitution of action ai with action aj if the user does action aj instead of ai.
- H4= {*swap* (ai,aj ): ∀ai,aj ∈ A}: Swapping of two actions if the user does the actions in reverse order.

The algorithm for identifying modifications is based on the constructing of *distance matrix*. D=[dm,n]|C|×|ρ_ij_|, is constructed as in Textbox 2

**Constructing Distance Matrix.**

**Algorithm**: constructDistanceMatrix (C, ρij )

**Input**: C and ρij

**Output**: distance matrix (D) and number of modifications

for m=0 to m=|C|

   for n=0 to n=|ρij|

      if C(m) == ρij(n) then

         dm,n=dm−1,n−1 // no modification needed

      else

         dm,n=minimum(

            dm−1,n + 1 // insertion

            dm,n−1 + 1 // deletion

   dm−1,n−1 + 1 // substitution

            if((C(m − 1) == ρij(n − 2))&(C(m − 2) == ρij(n − 1)) then

               dm−2,n−2 + 1 // swap )

   return D, d|C|,|ρ_ij_|

The number of modifications is given by the value of d|C|,|ρ_ij_|. In addition, the construction of the distance matrix allows to identify the set of modifications Mij = {mij_k_ : mij_k_ ∈ H}. For each value, the distance matrix records what modification(s) has been considered. The set of modifications is identified using the algorithm identifyModifications (D, |C|, |ρij|) (see Textbox 3.

**Identifying Modifications Based on Distance Matrix.**

**Algorithm**: identifyModifications (D, m, n)

**Input**: D and H

**Output**: set of modifications Mij

if (*m, n*)! = (0, 0) then

   if modificationsToConsider (dm,n) == empty then

      modificationsToConsider (D, m − 1,n − 1)

   if modificationsToConsider (dm,n) == H1 then

      Mij.add(insertion);

      modificationsToConsider (D, m − 1,n)

   if modificationsToConsider (dm,n) == H2 then

      Mij.add(deletion);

      modificationsToConsider (D, m, n − 1)

   if modificationsToConsider (dm,n) == H3 then

      Mij.add(substitution);

      modificationsToConsider (D, m − 1,n − 1)

   if modificationsToConsider (dm,n) == H4 then

      Mij.add(swap);

      modificationsToConsider (D, m − 2,n − 2)

return Mij

**Figure 3:**
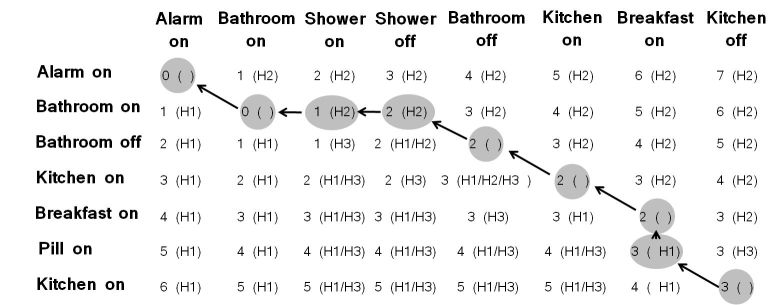
Generated distance matrix and identification of the set of modifications.

For example, the distance matrix for Michael’s current behavior C2 and his frequent behavior ρ11 is given by Figure 3. In this case, 3 modifications are needed, specifically deletion (‘Shower, on’), deletion (‘Shower, off’) and insertion (‘Pill, on’). Figure 3 shows how the set of modifications is identified.

The current behavior C2 needs only one modification, insertion (‘Pill, on’), in order to match the path ρ12. The current behavior C3 requires 3 modifications for the path ρ11, while it needs only 1 modification, swap (‘Pill, on’, ‘Breakfast, on’), for the path ρ12.

##### Identifying Criticality

The importance of each modification is different. In Michael’s example, the consequence of not taking the pill is far more important than forgetting to take the shower. Experts also advised him not to take the pill before having breakfast, whereas some other swaps could have no consequences. Depending on each environment and the knowledge collected from experts, relatives, etc, a criticality value can be assigned to each possible modification. Let g define a mapping from set H to a set of all possible criticality values V={vi = g(hi): ∀hi ∈ H}. Then, the criticality for a set of modifications, Mij, is obtained as in Equation 3 in Multimedia Appendix 1.

For example, given the criticality mappings (the lower the value, the more critical) shown in Multimedia Appendix 1, we have Cr(M12)=0.2 and Cr(M12)=0.4 for the current behaviors C2 and C3, respectively. For a set of observed actions, C, the algorithm can consequently determine the behavior risk factor defined as in Equation 4 in Multimedia Appendix 1, for all fi ∈ F. If φi is greater than a certain threshold, the current behavior C is declared as an anomalous behavior.

## Experimental Results and Discussion

This algorithm was validated using datasets collected from the Washington State University (WSU) Smart Apartment environment [20]. Data collected in the WSU Smart Apartment represent participants performing the same five ADLs (Activities of Daily Living) in the apartment: make a phone call, wash hands, cook, eat, and clean. Inside, there were motion sensors, cameras, and computers to control a variety of tasks, such as opening blinds or turning up heat or air conditioning. For example, there was automated computer software in the kitchen that handily provided a needed recipe and kept track of ingredients needed for recipes. Although the participants did not have Alzheimer’s disease and had to perform the same five ADLs, the performed set of actions could vary depending on each participant. The actions involved in each one of the activities are shown in Table 1.

**Table 1:** Actions involved in each ADL.

Activity	Involved Actions
Make a phone call	‘PhoneBook On’ →‘Phone On’ → ‘Phone Off’
Wash hands	‘Water On’ → ‘Water Off’
Cook	‘Cabinet On’ → ‘Raisins On’ → ‘Oatmeal On’ → ‘MeasuringSpoon On’ → ‘Bowl On’ → ‘Sugar On’ → ‘Cabinet Off’ → ‘Water On’ → ‘Water Off’ → ‘Pot On’ → ‘Burner On’ → ‘Burner Off’
Eat	‘Cabinet On’ → ‘Medicine On’ → ‘Cabinet Off’ → ‘Water On’ → ‘Water Off’ → ‘Cabinet On’ → ‘Medicine Off’ → ‘Cabinet Off’
Clean	‘Water On’ → ‘Water Off’

In order to validate the proposed algorithm, an adapted 10-fold cross validation process is performed. A simple 10-fold cross validation validates the frequent behaviors obtained using LFPUBS algorithm. Adapted cross-validation validates if the algorithm identifies shifts from frequent behaviors. As a result of this validation, carried out in an offline way, it has to be said that it was able to identify in all the cases (100%) if it matched a path, and its correspondent likelihood. When it did not match a frequent behavior, the algorithm was able to identify necessary modifications in all the cases (100%).

**Figure 4:**
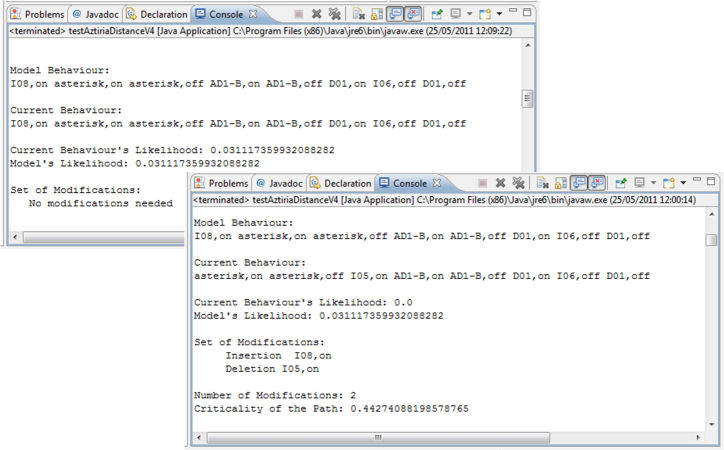
Example of outputs obtained in the validation process.

Figure 4 shows how results are provided by the system. On one hand, it shows a first example where the current behavior matches a path of the model, so that it does not need any modification. On the other, a current behavior that is not covered by the model is shown. In that case, necessary modifications were calculated (two modifications in this case) and the importance of those modifications is provided by the criticality parameter.

It is quite difficult to compare to other approaches due to the lack of shift detection approaches. Compared to activity recognition approaches, being able to identify all shifts is a very good result.

### Conclusion

Intelligent environments suggest a new paradigm where environments adapt their behaviors based on preferences and habits of users instead of the other way around. For that, environments must learn users’ frequent behaviors. But, at the same time, users will not always behave in accordance with those patterns. Users typically modify their habits over time, but some other factors (eg, age-related diseases) may influence the behavior.

This paper developed an algorithm that compares users’ current behaviors with their frequent behaviors and identifies possible shifts. If users’ current behavior is frequent, the algorithm determines its frequency. Otherwise, the algorithm identifies the shifts. Generally, such shifts can be used to adapt patterns. These shifts can also be used to detect early signs of diseases. In this case, the algorithm determines the criticality of a shift.

The algorithm was tested using a dataset collected in the WSU Smart Apartment. The algorithm showed the capacity to identify if a behavior was frequent or not as well as to identify the shifts.

As future work, we plan to extend this algorithm to an online algorithm in order to analyze behaviors online, so that reminders (eg, reminding the user to take a pill) can be issued if critical deviations or abnormal behaviors are identified. Adaptation of patterns based on deviations will also be addressed.

## Multimedia Appendix 1

Equations and mapping.

## Abbreviations

AAL: Ambient Assisted Living
ADL: Activities of Daily Living
AmI: Ambient Intelligence
LFPUBS: Learning Frequent Patterns of User Behavior
WSU: Washington State University
