# Supplementary material for: User Behavior Shift Detection in Ambient Assisted Living Environments
Source: JMIR Mhealth Uhealth. 2013 Jun 18;1(1):e6. doi: 10.2196/mhealth.2536 (PMC4114411; doi:10.2196/mhealth.2536)
Supplement: Supplementary file 1 [file mhealth_v1i1e6_app1.pdf]

Multimedia Appendix 1. Equations.

Equation 1.

$$LL\left(C|f_i\right) = \prod_{k=1}^{|C|-1} Pr\left(c_k \rightarrow c_{k+1}\right)$$

Equation 2.

$$LL(\rho_{ij}) = \prod_{k=1}^{|\rho_{ij}|-1} Pr\left(\rho_{ij_k} \rightarrow \rho_{ij_{k+1}}\right)$$

Equation 3.

$$Cr(M_{ij}) = \prod_{k=1}^{|M_{ij}|} g\left(M_{ij_k}\right)$$

Mapping

insert (*shower*, *on*) $\overset{g}{\rightarrow}$0.9 insert (*shower*, *off*) $\overset{g}{\rightarrow}$0.9

insert (*pill*, *on*) $\overset{g}{\rightarrow}$0.2 swap ((*breakfast*, *on*), (*pill*, *on*)) $\overset{g}{\rightarrow}$0.4

Equation 4.

$$\phi_i = \max_{\rho_{ij}} \frac{LL(\rho_{ij})}{Cr(M_{ij})}$$
